# Pre-service medical education course completion and drop-out rates

**DOI:** 10.1186/s12960-022-00785-2

**Published:** 2022-12-27

**Authors:** Osahon Enabulele, Joan Emien Enabulele

**Affiliations:** 1 Department of Family Medicine, University of Benin Teaching Hospital, Ugbowo, P.O. Box 10427, Benin City, Edo Nigeria; 2 Department of Family Medicine, University of Benin, Benin City, Edo Nigeria; 3 Department of Restorative Dentistry, University of Benin, P.M.B. 1154, Benin City, Edo Nigeria

**Keywords:** Course completion rate, Course drop-out rate, Retention, Education, Medical students, Training

## Abstract

**Introduction:**

The “Global strategy on human resources for health: Workforce 2030” was adopted by the 69th World Health Assembly. Among its objectives is the strengthening of data on human resources for health, to inform evidence-based policy decisions. These data include the course completion and drop-out rates, to inform mechanisms that support recruitment and retention.

**Objective:**

This paper sought to evaluate trends in course completion and drop-out rates of health workforce students. However, original data were only obtained for pre-service medical students, but no other health worker occupational groups.

**Methods:**

A mixed method approach was employed to obtain data presented in this paper. A structured questionnaire was sent out to targeted medical training institutions, regulatory bodies, and National Medical Associations, supplemented by a web and literature search for existing studies or data reports. Data were analyzed using IBM SPSS Statistics version 21.0 (Chicago, IL, USA) and Microsoft Excel 2010.

**Results:**

Eight previously published studies were identified originating from six countries, with course completion rates ranging from 84% in Pakistan to 98.6% in the United States of America, while the drop-out rates ranged from 1.4% in the United States of America to 16% in Pakistan. An analysis of pre-service medical students in Australia and New Zealand, revealed average course completion rates of 93.3% and 96.9%, respectively, and average drop-out rates of 6.7% and 3.1%, respectively. An analysis of pre-service medical students from Nigeria, revealed an average course completion rate of 88.3%, and an average drop-out rate of 11.7%. Data were not readily available for most countries targeted during the research, either because of lack of existing mechanisms for collation of required data or restrictions making such data publicly unavailable and inaccessible.

**Conclusions:**

Drop-out rate for pre-service medical students varies across countries with some countries recording higher drop-out rates, which raise significant concerns about the capacity of such countries to scale up production of human resources for health. Data that monitor both course completion and drop-out rates, and seek to provide insight into reasons for observed numbers, can inform mechanisms to address the causes of course drop-out and support student retention.

## Introduction

A health system consists of all the organizations, institutions, resources and people whose primary purpose is to improve health [1]. Among these components of a health system, Human Resources for Health (HRH) is critical towards the attainment of effective, equitable, accessible, affordable and quality health service coverage.

Despite the vital importance of HRH, the available HRH in most countries is unable to match population health needs. Estimates by the World Health Organization in 2016 projected a global shortage of 18 million health workers needed by 2030 to achieve the Universal Health Coverage (UHC) targets of the Sustainable Development Goals (SDG) 2030 agenda [2]. This deficit in HRH cuts across various countries of the world, with countries at all levels of development contending with varying levels and forms of HRH challenges, such as education/production, deployment, maldistribution and inefficient use of HRH, and poor working conditions [2]. This is worsened by the unceasing emigration of health workers, particularly from low- and middle-income (LMIC) countries where the challenge of HRH shortage is even more critical [3, 4]. The challenge is also compounded by the general dearth of health workforce data for planning, decision-making and tracking of progress in HRH in various countries.

It was in realization of the need to address this gap in HRH, through stimulation of greater political commitment to health financing and investment in the education, recruitment, training, development and retention of the health workforce, particularly in developing countries, that the “Global Strategy on Human Resources for Health: Workforce 2030” was adopted by the 69th World Health Assembly [2]. The strategy has as its vision the acceleration of progress towards Universal Health Coverage and the United Nations Sustainable Development Goals through equitable access to health workers within strengthened health systems. Among its four core objectives is the strengthening of data on human resources for health, for monitoring and ensuring accountability for the implementation of national, regional, and global strategies.

The foregoing underscores the basis for this paper which seeks to appraise data on graduation/course completion rates and drop-out rates of medical students from medical educational and training institutions, with the hope of providing insights to inform planning and decision-making on medical workforce production, recruitment and retention.

For the purpose of this paper, drop-out rate is defined as the rate of students from a cohort of students exiting a health workforce education and training program without completion [5], while graduation/ course completion rate is defined as the ratio of the number of students graduating from a health workforce education and training program to the number of students enrolled in the first year of the same education and training program [5].

## Methods

A mixed method approach was employed to obtain data presented in this paper.

A structured questionnaire was sent to targeted medical training institutions and regulatory bodies, as well as National Medical Associations that are members of the Commonwealth Medical Association. This was done through direct contact with the medical training institutions and regulatory bodies, as well as through direct contacts with National Medical Associations in fifteen (15) countries, distributed across Africa, Asia, the Caribbean, Europe and America. The structured questionnaire consisted of a section which highlighted the purpose of the study, the definition of terms to be studied, as well as assurance of confidentiality of the data to be obtained from the respondents. It also had a section which sought to elicit data on the number of medical students admitted and the number of medical students that graduated over a 12-year period (between the year 2009 and 2020). The websites of some medical schools and national medical regulatory bodies and associations, were also accessed for relevant information and data. This included publicly available secondary data sourced from the website of Medical Deans in New Zealand and Australia [6]. Additionally, a primary search and review of peer-reviewed literature and studies on the subject was conducted, using PubMed and Google scholar databases. Search terms used for the literature search included: Medical, School attainment, Medical Student progression, Medical Student retention, Course completion rate, Attrition rate, Drop-out rate, Failure rate.

Obtained data were analyzed using IBM SPSS Statistics version 21.0 (Chicago, IL, USA) and Microsoft Excel 2010. Descriptive statistics were deployed and results were presented as percentages, rates, and graphs.

## Results

Eight previously published studies were identified from the literature search, originating from six countries as shown in Table 1: United States of America (*n* = 2), United Kingdom (*n* = 2), Netherlands (*n* = 1), Pakistan (*n* = 1), Saudi Arabia (*n* = 1), and Germany (*n* = 1). The course completion rates in the published studies ranged from 84% in Pakistan to 98.6% in the United States of America, while the drop-out rates ranged from 1.4% in the United States of America to 16% in Pakistan (Table 1).

**Table 1:** Descriptions of included studies

Author(s)	Year of publication	Country	Course completion rate	Drop-out rate
Heublein et al. [13]	2020	Germany	90.0%	10.0%
AAMC et al. [8]	2018	USA	95.9%	4.1%
Maher et al. [12]	2013	Netherlands	94.3	5.7
Gillian et al. [14]	2009	UK	90.0%	10.0%
Al-Mazrou [11]	2008	Saudi Arabia	96.2%	3.8%
Gwen et al. [10]	2007	USA	98.6%	1.4%
Huda et al. [16]	2004	Pakistan	84.0%	16.0%
Simpson et al. [15]	1996	UK	86.0%	14.0%

**Fig. 1:**
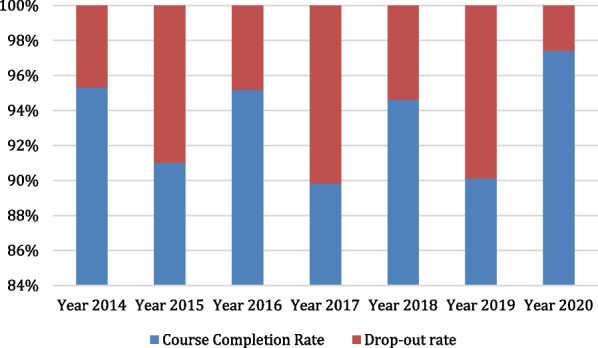
Course completion and drop-out rates for pre-service medical students in New Zealand (2014–2020)

From the website of Medical Deans in New Zealand and Australia [6], some publicly available secondary data were sourced and analyzed. From an analysis of 7 sets of pre-service medical students in New Zealand, this paper found that whereas the course completion rates ranged from 89.8 to 97.4% with an average course-completion rate of 93.3%, the drop-out rates ranged from 2.6 to 10.2% with an average drop-out rate of 6.7% (Fig. 1).

**Fig. 2:**
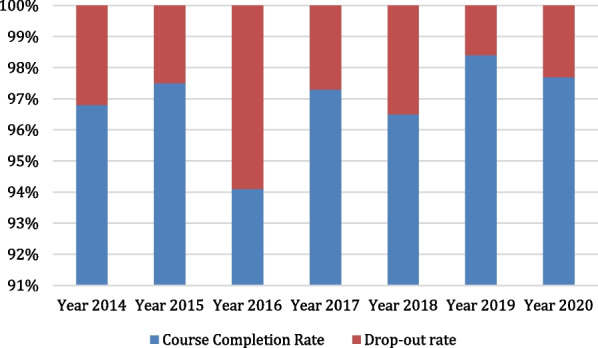
Course completion and drop-out rates for pre-service medical students in Australia (2014–2020)

Similarly, from an analysis of 7 sets of pre-service medical students in Australia, this study found that the course completion rates ranged from 94.1 to 98.4% with an average course-completion rate of 96.9%, while the drop-out rates ranged from 1.6 to 5.9% with an average drop-out rate of 3.1% (Fig. 2).

Three additional datasets were identified but not publicly accessible. The United Kingdom Medical Education Database (UKMED) [7] offers a biannual cycle of data release for datasets matched within confidentiality legal requirements, but the dates were not substantially within the study period (October–December, 2021); the Association of American Medical Colleges (AAMC) Student Records System (SRS) [8] is a confidential, password-protected, secure, Internet-based records system developed for use by medical school registrars and their designated authorized users; and the database of the Organization for Economic Co-operation and Development (OECD) developed for use by authorized users [9].

**Fig. 3:**
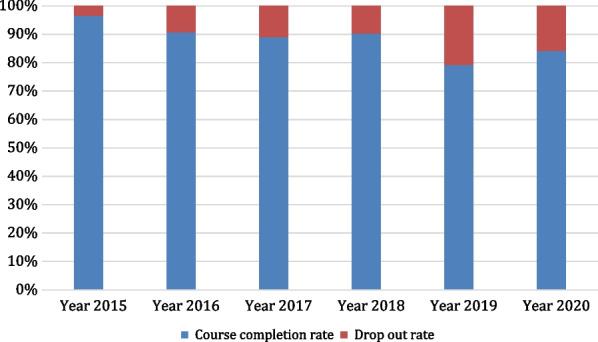
Course completion and drop-out rates of pre-service medical students in Nigeria (2015–2020)

Only primary data from the Medical and Dental Council of Nigeria were received in response to the questionnaire. The data collected in Nigeria were for the period 2015–2020, upon completion/graduation by the medical students. The data collected were to be compared with schools worldwide across the same period. An analysis of the data revealed a course completion rate that ranged from 79.2% in the year 2019 to 96.5% in the year 2015, with an average course completion rate of 88.3%, while the drop-out rate ranged from 3.5% in the year 2015 to 20.8% in the year 2019, with an average drop-out rate of 11.7%. Drop-out rate was observed to be highest in 2019 (Fig. 3).

## Discussion

The “Global strategy on human resources for health: Workforce 2030” was adopted by the 69th World Health Assembly. Amongst its objectives is the strengthening of data on human resources for health. These data include the course completion and drop-out rates which are important indices that should be of concern to medical education and training institutions, and to WHO Member States, to inform mechanisms that support recruitment and retention.

Aside from the health workforce shortages seen in most countries, worsened by the phenomenon of emigration, course completion and drop-out rates among medical students and students in allied health professions, have significant economic impact. A high drop-out rate can lead to significant economic losses to a country, as the public investments made on the education and training of students before they dropped out, are wasted. Apart from the fact that high drop-out rates may negatively affect the image of the affected institutions, the affected students and their families may suffer psychosocial problems of rejection and reduced self-worth, as well as the opportunity cost and financial investments made in their own studies.

Whereas the education of various cadres of health professionals through existing health educational and training institutions remains a fundamental factor in the development of HRH, there exist limited data on the course completion and drop-out rates of medical students and students in professions allied to medicine. Though this study sought data across health professional and health workforce groups, only data for medical education/ pre-service medical students were obtained. Unlike what was found in the case of New Zealand, Australia, United States of America (USA), and the United Kingdom (UK), with platforms for data collation and tracking of the performance of medical students, this study found a substantial lack of effective and efficient platforms and mechanisms for such data collation in most institutions and countries from which data were sought. Whereas little or no difficulty was experienced in accessing secondary data from New Zealand and Australia [6], the limited period for this study coupled with the fact that data were not publicly accessible in some countries due to some restrictions and requirements for application for data (sometimes within a narrow window period for application) made it difficult to access secondary data from the United Kingdom Medical Education Database (UKMED) [7], the database of the Association of American Medical Colleges (AAMC) Student Records System (SRS) [8], and the database of the Organization for Economic Co-operation and Development (OECD) [9].

It is apparent from available literature and analyzed data that the course completion and drop-out rates of medical students vary across countries. Data are unavailable to determine whether there is further variation within countries, or within or across occupations. An analysis of publicly available data on pre-service medical students sourced from Medical Deans in New Zealand [6], between the year 2014 and the year 2020, revealed relatively stable high course completion and low drop-out rates. Similarly, a study of publicly available data on pre-service medical students sourced from Medical Deans in Australia [6] between the year 2014 and the year 2020 also revealed relatively stable high course completion and low drop-out rates, even though the rates for Australia were relatively more stable than those for medical students in New Zealand. Some drop-out rates are to be expected; this could be due to personal life choices, health reasons, insufficient academic progression or fitness to practice reasons. The reasons for the variations found in the course-completion and drop-out rates among pre-service medical students in New Zealand and Australia are not immediately known. In the United States of America, a study conducted among three (3) cohorts of matriculating classes of medical students and followed up for 10 years each, revealed a relatively high graduation/course completion rate and a low drop-out rate. Among all the medical students across the 3 cohorts, it was reported that only 1.4% of the medical students left medical school [10]. This study finding is similar to the findings in another study on course completion and drop-out rates of US medical students, sourced from the website of the Association of American Medical Colleges (AAMC) Student Records System (SRS) [8]. The AAMC study found that the medical school graduation rates for students undergoing the Doctor of Medicine (MD) medical degree program remained stable from 1993–1994 through 2012–2013, with a 4-year graduation rate that ranged from 81.6 to 83.4% and with a total national attrition rate of 3.3%. It also found that 6 years after matriculation, the average graduation rate was 95.9% for MD students not participating in combined degree programs. This amounts to a drop-out rate of 4.1%. These findings are similar to findings from a 2004 cross-sectional study of students admitted into the College of Medicine, King Saud University, Saudi Arabia. A study of 5 academic years (1994–1998) revealed a low drop-out rate of 3.8% [11]. Similarly, a retrospective descriptive study of medical school attrition over a 10-year period (2001–2011) carried out at the University College Cork, revealed an overall drop-out rate of 5.7% [12], while another study conducted by Heublein et al. [13] on drop-out rate among medical students in Germany found a drop-out rate of 10%. Unlike the drop-out rate of about 10.0% and a high course completion rate (about 90.0%) that were found in a comparative study conducted among 5-year undergraduate and 4-year graduate entry medical students who graduated in the year 2007 and 2008 from the University of Nottingham, United Kingdom [14], a drop-out rate of 14% was found from the result of a retrospective study of records of medical students between 1983 and 1992 at Leeds School of Medicine, United Kingdom [15]. The variation in the UK studies may be due to the varied number of cohorts, categories, and number of medical students that were studied, and different interventions introduced over the time period. A previous study of 396 medical students in Pakistan conducted over a 6-year period (1996–2001), revealed a drop-out rate of 16%. [16]

While it was difficult to source primary and secondary data from low- and middle-income countries (LMIC) largely due to non-existing institutional platforms for regular collation of such important data, data on medical students from medical schools in Nigeria were sourced through the platform of the Medical and Dental Council of Nigeria (MDCN) which regulates the medical and dental professions in Nigeria [17]. The obtained data revealed a contrast from the data obtained from New Zealand and Australia with an average course completion rate of 88.3%, and an average drop-out rate of 11.7%. When the findings from New Zealand and Australia are compared with the findings from Nigeria, we appreciate the variations in course completion and drop-out rates between countries. In comparison with the rates obtained for New Zealand and Australia, the observed higher drop-out and lower course-completion rates among medical students in Nigeria, particularly in the respective years of 2019 (drop-out rate of 20.8%) and 2020 (drop-out rate of 15.8%) is worthy of further interrogation. The recorded higher drop-out rates may however be attributed to periods of stagnation in some medical schools in Nigeria due to suspension of the accreditation status of some medical schools, disruptions in the academic calendar caused by frequent industrial actions by academic staff of universities, industrial actions by medical doctors and other health workers, as well as disruptions due to the COVID-19 pandemic (particularly during the first wave of COVID-19 in Nigeria in the year 2020) [18–23]. The difference in admission policies, duration of study, curriculum, and adopted teaching and training methods may also account for the difference in drop-out rates between the countries [24] as the traditional type of curriculum is used in Nigeria. Furthermore, as was observed in the literature review done by Arulsamy Anand [25] on reasons for drop-out in medical schools, other probable reasons for the high drop-out rate may be the lack of motivation and weak academic abilities of some medical students, health challenges, and financial constraints experienced by some medical students (especially those with poor socio-economic backgrounds) worsened by absence of student loans and bursaries. Another factor may be the availability of student welfare services including academic advising and counselling services, as well as the increasing emigration of families from Nigeria to other developed countries, with medical students withdrawing from school to enable them emigrate with their families. [26, 27]

### Limitations

There was great difficulty in accessing data on course completion and drop-out rates from medical educational training institutions/medical schools, essentially due to the virtual non-existence of institutional platforms and mechanisms for such data collation, and in some cases, the restrictions imposed on public access to such data. These significantly limited the data that could be sourced directly in the course of this study. Another limitation was the fact that majority of available literature on course completion and drop-out rates involved studies conducted in high income and developed countries, a fact that made comparative analysis difficult. The non-uniform number and cohort of medical students studied in this paper and in most of the literature found on course completion and drop-out rates, as well as the limited number of countries studied, is a limitation on the generalizability of the study findings. Another limitation was the non-segregation into public and private medical schools in most of the publicly available data. A segregation of the data would have helped to evaluate if there were any variations in the course completion and drop-out rates between public and private medical schools.

## Conclusions

To date, the majority of published studies about drop-out rates of medical students are focused on high income, stable countries; to our knowledge, this is the first availability of comprehensive national data on drop-out rates from Nigeria. In the context of efforts to scale up and sustain a strong health workforce to achieve Universal Health Coverage, it is concerning to find drop-out rates of more than 10%, as was reported in some of the studied countries. The importance of accurate recording of students' matriculation and graduation by institutions providing medical education to inform workforce planning cannot be overemphasized.

Therefore, it is recommended that countries and medical educational training institutions develop mechanisms for seamless and regular data collection/collation and archiving on attrition/drop-out and completion rates. Accrediting bodies for medical schools should include publication of attrition/drop-out and completion rates as requirement for accreditation. Secondly, access must be ensured to enable interpretation from this data; this may include securing mechanisms to match or collate datasets before releasing for research analysis, as is the practice with UKMED. Access to these data will facilitate planning and informed policy decision-making on strategies for improving HRH. Efforts should also be made to make data on medical education easily accessible without violating confidentiality. Further studies on health workforce education course completion and drop-out rates should be undertaken to provide further insights and to address observed limitations of this study.

## Data Availability

The datasets used and/or analyzed during the study are available from the corresponding author on reasonable request.

## Abbreviations

AAMC: Association of American Medical Colleges
HRH: Human Resource for Health
LMIC: Low- and middle-income country
MD: Doctor of Medicine
MDCN: Medical and Dental Council of Nigeria
SDG: Sustainable Development Goal
SRS: Student Records System (SRS)
UHC: Universal Health Coverage
UK: United Kingdom
UKMED: United Kingdom Medical Education Database
USA: United States of America
WHO: World Health Organization
